# Monitoring and evaluation of vegetation restoration in the Ebinur Lake Wetland National Nature Reserve under lockdown protection

**DOI:** 10.3389/fpls.2024.1332788

**Published:** 2024-04-18

**Authors:** Nan Xia, Yuqian Tang, Mengying Tang, Weilin Quan, Zhanjiang Xu, Bowen Zhang, Yuxuan Xiao, Yonggang Ma

**Affiliations:** ^1^ College of Geographical and Remote Sensing Sciences, Xinjiang University, Urumqi, China; ^2^ Xinjiang Key Laboratory of Oasis Ecology, Xinjiang University, Urumqi, China; ^3^ Key Laboratory of Smart City and Environment Modelling of Higher Education Institute, Xinjiang University, Urumqi, China; ^4^ College of Ecology and Environment, Xinjiang University, Urumqi, China

**Keywords:** Ebinur Lake Wetland National Nature Reserve, geographical detector, multisource remote sensing, NDVI, vegetation trend

## Abstract

For a long time, human activities have been prohibited in ecologically protected areas in the Ebinur Lake Wetland National Nature Reserve (ELWNNR). The implementation of total closure is one of the main methods for ecological protection. For arid zones, there is a lack of in-depth research on whether this measure contributes to ecological restoration in the reserve. The Normalized Difference Vegetation Index (NDVI) is considered to be the best indicator for ecological monitoring and has a key role to play in assessing the ecological impacts of total closure. In this study, we used Sentinel-2, Landsat-8, and Moderate Resolution Imaging Spectroradiometer (MODIS) remote sensing data to select optimal data and utilized Sen slope estimation, Mann-Kendall statistical tests, and the geographical detector model to quantitatively analyze the normalized difference vegetation index (NDVI) dynamics and its driving factors. Results were as follows: (1) The vegetation distribution of the Ebinur Lake Wetland National Nature Reserve (ELWNNR) had obvious spatial heterogeneity, showing low distribution in the middle and high distribution in the surroundings. The correlation coefficients of Landsat-8 and MODIS, Sentinel-2 and MODIS, and Sentinel-2 and Landsat-8 were 0.952, 0.842, and 0.861, respectively. The NDVI calculated from MODIS remote sensing data was higher than the value calculated by Landsat-8 and Sentinel-2 remote sensing images, and Landsat-8 remote sensing data were the most suitable data. (2) NDVI indicated more degraded areas on the whole, but the ecological recovery was obvious in the localized areas where anthropogenic closure was implemented. The ecological environment change was the result of the joint action of man and nature. Man-made intervention will change the local ecological environment, but the overall ecological environment change was still dominated by natural environmental factors. (3) Factors affecting the distribution of NDVI in descending order were as follows: precipitation > evapotranspiration > land use type > elevation > vegetation type > soil type > soil erosion > slope > temperature > slope direction. Precipitation was the main driver of vegetation change in ELWNNR. The synergistic effect of the factors showed two-factor enhancement and nonlinear enhancement, and the combined effect of the driving factors would increase the influence on NDVI.

## 1 Introduction

The Ebinur Lake Wetland National Nature Reserve (ELWNNR) is a typical representative of inland lake wetlands in Xinjiang. ELWNNR is rich in salt mines, *Artemia parthenogenetica*, *Cistanche deserticola*, and shrub forests (Zhang et al., 2021a). For the management of the protected area, the state stipulates that no human activities should occur in its core. However, we learned from the local forestry department that before 2017, ditch digging, brine worm fishing, and aquaculture were still occurring in the core area of ELWNNR, which may have destabilized the local ecosystem. In 2017, China carried out a comprehensive environmental protection inspection of protected areas, including ELWNNR, which required conducting ecological restoration work as soon as possible and prohibiting all human and animal activities in the core area. Under the supervision of several departments, including forestry, natural resources, and ecology, production and living in ELWNNR were completely banned. Generally, the protection methods for nature reserves include expansion, promotion, and closure, among which closure protection is a management method that excludes all human activities and maintains the original ecology of the reserve, which has the advantages of conforming to the laws of nature and is easy and inexpensive to manage (Peng et al., 2006). However, local scholars have learned that appropriate human production and living activities can help in local ecological recovery. Such activities include catching brine worms and collecting salt minerals, which can purify the water quality and improve the water environment, thus aiding the growth of vegetation. Scholars are more in favor of maintaining a balance between economic development and ecological protection. Therefore, whether the implementation of total closure protection is effective for the ecological recovery of ELWNNR is worth exploring.

Vegetation is an important part of terrestrial ecosystems, and its growth can reflect the quality of the regional ecological environment, so monitoring vegetation is a reliable means to evaluate the effectiveness of closure protection. The normalized difference vegetation index (NDVI) can reflect the growth of vegetation, and it is the best indicator for evaluating vegetation coverage and monitoring the ecological environment (Gao et al., 2021). NDVI is widely used in arid zones. Potential relationships between vegetation and anthropogenic drivers can be identified through dryland ecosystem testing in northwestern Ethiopia (Worku et al., 2017). Rising temperatures and evapotranspiration in Asia were major factors contributing to the greening of vegetation in northeastern and central China (Lamchin et al., 2018). In terms of wetland ecosystems, a number of scholars have conducted research on ecological restoration on the basis of NDVI. At the Ebinur Lake watershed, quantitative evaluation of vegetation dynamics can characterize the dynamics of the surface (Zhang et al., 2020). In the study of monitoring vegetation changes in alpine wetlands on the Tibetan Plateau, some scholars explored the dynamic changes of NDVI in wetlands and its correlation with temperature, precipitation, and solar radiation (Chen et al., 2023). In exploring the long-term spatiotemporal pattern of vegetation change in the Dongting Lake wetland and its response relationship to climate change and human activities, human activities promoted the restoration of wetland vegetation, and climate change threatened the wetland vegetation (Zhang et al., 2021b). In the study of vegetation cover change and migratory bird distribution in the Poyang Lake wetland, the correlation coefficients between vegetation and different types of migratory birds were obtained by NDVI as an indicator to reveal the change in wetland vegetation (Wu et al., 2014). Satellite remote sensing observation technology, with its unique advantages, provides data on different spatial scales for monitoring vegetation growth, which helps assess climate change and anthropogenic changes in vegetation. Currently, abundant and diverse remote sensing datasets are available in the international arena, including considerable data suitable for calculating the NDVI. Compared with other remote sensing datasets, Sentinel-2, Landsat-8, and Moderate Resolution Imaging Spectroradiometer (MODIS) remote sensing data have different spatial, temporal, and spectral resolutions; specifically, they have finer spatial and temporal resolutions (Shen et al., 2021). They can better reflect vegetation growth from multiple spatial resolutions, temporal perspectives, and directions and can provide diverse information for monitoring vegetation cover and health. Scholars have examined the surface area of Ebinur Lake through Landsat and Sentinel data and explored the drivers of its dynamic changes (Wang et al., 2019a). The detection of soil salinity in ELWNNR has been conducted to explore the difference between Landsat-8 and Sentinel-2 detection accuracy (Wang et al., 2020). However, studies on vegetation are lacking, and the three types of data have diverse spatial and temporal resolutions, with differences in the performance effects on sparse vegetation in arid zones. Therefore, selecting optimal data to reflect the sparse vegetation in arid zones on the ELWNNR scale is important for ecological quality assessment.

Vegetation changes can directly reflect changes in ecosystems and their impacts on the environment under the influence of anthropogenic and natural factors (Guo et al., 2021). Understanding not only the status of vegetation growth and change but also the drivers of vegetation change is important (Chang et al., 2022). In the context of global climate change, the analysis of vegetation status, driving factors, succession patterns, trend prediction, and overall regulation have become indispensable topics. Numerous scholars have proposed traditional research methods, such as principal component analysis (Mberego et al., 2013), trend analysis (Sandra et al., 2015), support vector machines (Su et al., 2007), random forests (Schonlau and Zou, 2020), and other models and algorithms to explore the diversity of the dynamic change and complexity of vegetation drivers. However, there is a key shortcoming in these methods: they assume a certain linear relationship between vegetation and drivers at the time of use, but a strict linear relationship does not necessarily exist in this transformation process. The geographical detector is a novel spatial statistical method that, independent of any assumption of linearity, can quantify the spatial heterogeneity of vegetation and its drivers, as well as the interactions between the factors (Wang and Xu, 2017). In arid and semi-arid zones, the effects of natural and human factors and their interactions on the spatial and temporal changes of vegetation cover in the Ebinur Lake Watershed can be investigated, and the suitable range of each influence factor to promote vegetation growth can be analyzed (Ren, 2022). Playing an important role in oasis-desert ecotone hydrological climate change and anthropogenic impacts on NDVI (Chang et al., 2022). Ecological restoration, through the evaluation of the impact of natural vegetation, can combat desertification and soil erosion (Wang et al., 2021b). The main drivers of spatial and temporal desertification changes in the Northwest Arid Zone can be analyzed based on multiple meteorological and spatial attribute datasets (Hua and Hao, 2021). Precipitation and land use were the main drivers influencing vegetation change in the Black River basin (Zhu et al., 2020). The effects of anthropogenic activities on NDVI in the oasis-desert interface zone were found to be more important than environmental factors in the vegetation changes at the southern edge of Dunhuang City and the northern side of Mingsha Mountain (Zhang et al., 2019). Therefore, the geographical detector has strong applicability and obvious advantages and has been widely used in arid and semi-arid zones.

Although scholars have analyzed the ecological changes in the Ebinur Lake watershed, evaluation of the effectiveness of ecological restoration management measures, especially excluding the impact of human activities (after 2017) on ecological restoration in the region, is lacking. Therefore, the objectives of our study were (1) to analyze the changes in vegetation dynamics in ELWNNR by comparing the optimal remote sensing data (Sentinel-2, Landsat-8, and MODIS remote sensing data) selected from 2016 to 2022; (2) to evaluate the spatial and temporal trends of NDVI, the trends of temperature, precipitation, and evapotranspiration, and the reasons for the changes in NDVI in the areas where human activities have existed and to discuss the effectiveness of closure and protection and the effectiveness of the restoration of the vegetation cover of ELWNNR; (3) to assess the driving force of the factors of ELWNNR vegetation changes and the effects of interactions. This study is a scientific reference for the multisource dynamic monitoring of vegetation change in ELWNNR, which is conducive to improving the continuity and accuracy of ecological restoration monitoring, evaluating the effectiveness of the implementation of comprehensive closure protection management measures, and providing a basis for research on the monitoring and protection of the ecological restoration of vegetation in other protected wetland areas.

## 2 Datasets and methods

### 2.1 Study area

**Figure 1:**
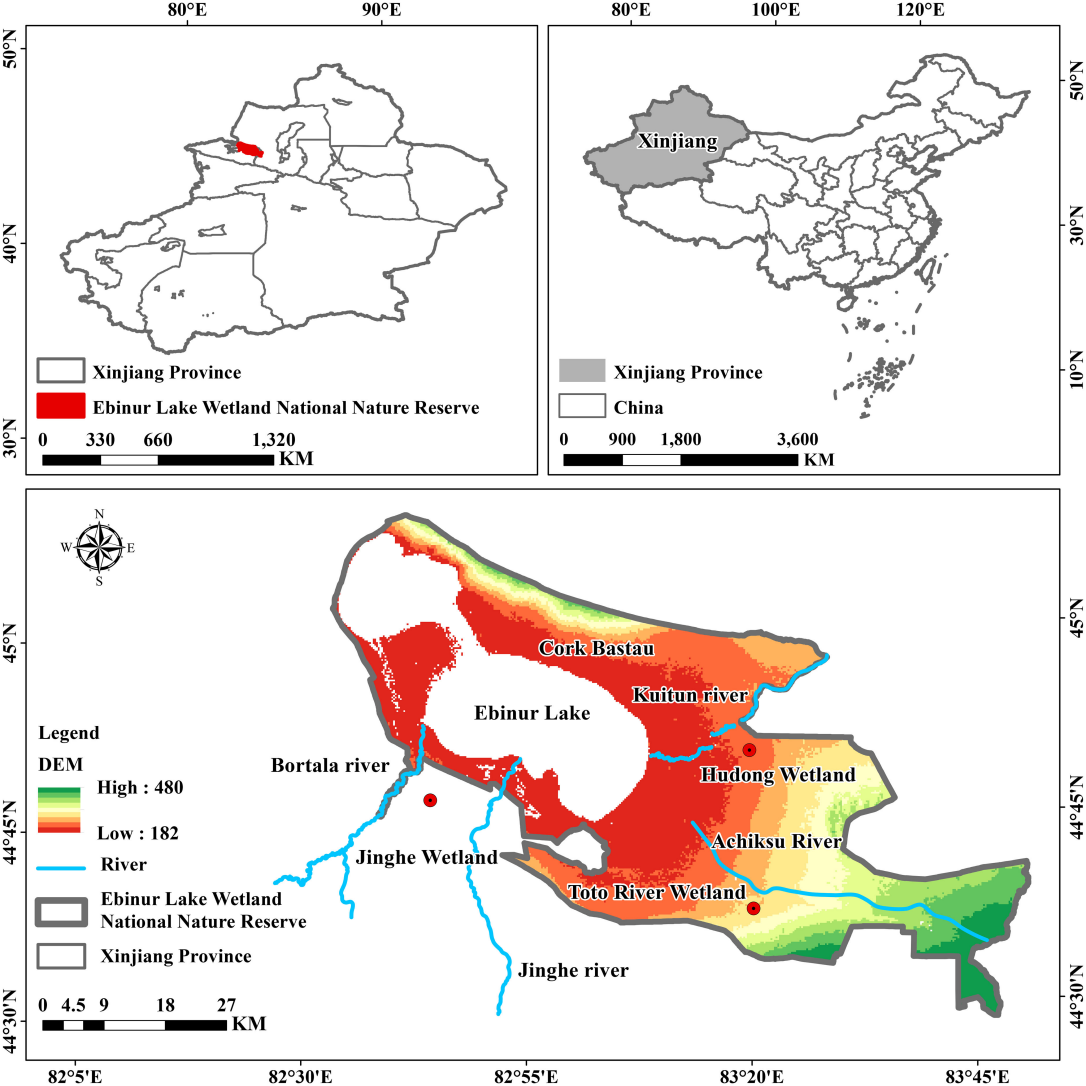
Location of the study area.

ELWNNR is located in the lowest depression at the southwest margin of the Junggar Basin in Xinjiang (82°36′–83°50′ E, 44°30′–45°09′ N) (Yue et al., 2017) with an area of 2670.85 km^2^ and is distributed with the Toto River Wetland and the Hudong Wetland (**Figure 1**). It has a temperate continental arid climate with abundant sunshine and a dry climate all year round, with an average annual precipitation of 105.17 mm, an average annual evaporation of 1,315 mm (Yang et al., 2009), and an average annual temperature of 5°C. ELWNNR contains the largest brackish water lake in Xinjiang, Ebinur Lake, which is the convergence center of surface water and groundwater from Bortala, Jinghe, Kuitun, and Achiksu rivers (Zhang et al., 2013) and is a closed inland lake. Soil types are mainly gray desert soils, gray brown soils, and wind-blown sand. The vegetation types are desert, meadow, and cultivated vegetation (Qin et al., 2016), primarily *Populus euphratica, Haloxylon ammodendron, Ebihuye, Tamarix chinensis, and C. deserticola* (Fu et al., 2009). In recent years, secondary salinization of soil has been widely distributed under the influence of the natural environment and human activities, posing a serious threat to ELWNNR and its surrounding ecological environment (Wang et al., 2019b).

### 2.2 Data source and preprocessing

From 2016 to 2022, when the vegetation was growing vigorously (from July to August) (Zhang et al., 2011), we conducted remote sensing observations of vegetation to gain a comprehensive understanding of vegetation growth in ELWNNR (**Table 1**). Landsat-8 images were obtained from the US Geological Survey (https://earthexplorer.usgs.gov/) and the Geospatial Data Cloud website (http://www.gscloud.cn/), column number 146, row number 29, and cloudiness less than 10%. The MODIS datasets were obtained from MOD13Q1-NDVI data provided by the NASA (National Aeronautics and Space Administration) Data Center (https://ladsweb.modaps.eosdis.nasa.gov/), with a total of 14 images. The MODIS ReProjection Tools were used to format and project the images, and the maximum value composite (MVC) method was used to convert the 16-day MODIS NDVI data into monthly average data. Sentinel-2 was provided by the ESA Data Center (https://scihub.copernicus.eu/dhus/#/home). Atmospheric corrections, resampling, and mosaicing were performed using Sen2cor and SNAP software (**Table 1**). Various types of data were also collected to determine the driving factors affecting the vegetation distribution. These included ten representative and easily quantifiable factors of elevation, slope, aspect, precipitation, temperature, evapotranspiration, land use type, vegetation type, soil type, and soil erosion. Elevation, slope, and aspect can change the hydrothermal conditions to change the vegetation distribution (Feng et al., 2021). Precipitation, temperature, and evapotranspiration were important factors directly affecting vegetation change (Pang et al., 2017). Vegetation type, soil type, and soil erosion were important environmental factors for vegetation growth (Peng et al., 2019b). Land use type can be used as an indicator of economic development and ecological change (Nie et al., 2021). The elevation data were downloaded from the Geospatial Data Cloud (http://www.gscloud.cn), and the slope and aspect were calculated from the elevation data. Soil type, vegetation type, and soil erosion data were obtained from the Chinese Academy of Sciences Resource and Environmental Science and Data Centre (https://www.resdc.cn/). Temperature, precipitation, and evapotranspiration data were obtained from the National Tibetan Plateau Science Data Center (https://data.tpdc.ac.cn/home) (Peng et al., 2019a). Land use data were obtained from Esri Land Use Data (Esri Land Cover (arcgis.com)). The above data were divided into corresponding categories according to the existing classification system (**Figure S1**). ArcGIS was utilized to create a fishing net, and 266 fishing net points of 3 km × 3 km were set up based on the scope of the study area, the spatial attribute values of each factor were extracted.

**Table 1:** Data sources used in the study.

Sensor	Spatial Resolution (m)	Temporal Resolution (days)	Coverage(years)
Sentinel-2	10, 20, 60	5	2016-2022
Landsat-8	30	16	2016-2022
MOD13Q1	250	16	2016-2022

### 2.3 Methods

#### 2.3.1 NDVI classification

In order to characterize the changes in vegetation growth dynamics more visually, we used ArcGIS to classify ELWNNR-NDVI into five classes according to the equidistant spacing method (Bai et al., 2019), which were: low vegetation coverage (<0.2), medium low vegetation coverage (0.2–0.4), medium vegetation coverage (0.4–0.6), medium high vegetation coverage (0.6–0.8), and high vegetation coverage (0.8–1.0).

#### 2.3.2 Sen slope estimation and Mann-Kendall trend test

The Sen slope estimate is insensitive to data measurement errors and is computationally efficient (Militino et al., 2020). Applicable for analyzing ELWNNR-NDVI trends with the expression (Liu et al., 2015). The (Equation 1) is as follows:


(1) $$\beta =median\left(\frac{ndvi_{j}-ndvi_{i}}{j-i}\right),1<i<j<n$$

Where β is the estimated value of the trend slope in the data series, β>0 indicates that the time series presents an upward trend, and β<0 indicates that the time series presents a downward trend.

The Mann–Kendall trend test is widely used in hydrology and meteorology to test trends in long time series data, does not require that the sample points follow a specific pattern, is not disturbed by outliers, and is suitable for testing the significance of trends in ELWNNR-NDVI changes (Li et al., 2022). Given the significance level ɑ = 0.05 in MATLAB, when the absolute value of Z is 1.65, 1.96, and 2.58 nodes, the trend of change is divided into 8 classes.

#### 2.3.3 Geographical detector

The geographical detector is a statistical method used to detect spatial heterogeneity and its driving factors (Wang and Xu, 2017). The relationship between the independent and dependent variables is more reliable than in classical regression, and colinearity between the independent variables is avoided. The optimal spatial discretization function of the “GD” package in R was used to implement the geodetector model.

(1) Factor detector: It is used to calculate the spatial heterogeneity of different factors and detects how much a certain factor X explains the spatial heterogeneity of attribute Y. The Equations 2–4 is as follows (Wang and Xu, 2017):


(2) $$q=1-\frac{\sum_{h=1}^{L}N_{h}\sigma_{h}^{2}}{N\sigma^{2}}=1-\frac{SSW}{SST}$$

(3) $$SSW=\sum_{h=1}^{L}N_{h}\sigma_{h}^{2}$$

(4) $$SST=N\sigma^{2}$$

In this formula, q is the explanatory power of the independent variable X on the dependent variable Y, with a value range of [0, 1]. The larger the q value, the more obvious the spatial heterogeneity and the stronger the explanatory power of X on Y. The study area is divided into h = 1, 2,…, L regions; Nh and N are the number of units in layer h and the whole region, respectively; σh^2^ and σ^2^ are the variances of the Y values of layer h and region, respectively; SSW and SST are the sum of variance within layer and total variance of region, respectively.

In this study, the independent variable X is denoted as X_i_ (i = 1, 2, 3, 4, 5, 6, 7, 8, 9, and 10), and the optimal grading method is selected to grade it (Liu and Li, 2017). The dependent variable Y is NDVI (**Table S1**).

(2) Interaction detection: It is used to detect the interaction between two different factors and to evaluate whether the two factors increase or decrease the explanatory power of Y when acting together or independently of each other (**Table S2**).

(3) Ecological detector: It is used to determine whether there is a significant difference between two factors. Equations 5, 6) is as follows (Wang and Xu, 2017):


(5) $$F=\frac{N_{X1}\left(N_{X2}-1\right)SSW_{X1}}{N_{X2}\left(N_{X1}-1\right)SSW_{X2}}$$

(6) $$SSW_{X1}=\sum_{h=1}^{L1}N_{h}\sigma_{h}^{2},\;SSW_{X2}=\sum_{h=1}^{L2}N_{h}\sigma_{h}^{2}$$

Where $N_{X1}$ and $N_{X2}$ represent the sample number of two factors, respectively. $SSW_{X1}$ , $SSW_{X2}$ represent the sum of intra-layer variance formed by two factors, respectively. L1 , L2 represent the number of stratifications of variables X1 and X2 , respectively.

(4) Risk detection: It is used to compare whether there is a significant difference between the mean values of the dependent variables in the two regions. The Equation 7 is as follows (Wang and Xu, 2017):


(7) $$t=\frac{{\bar{Y}}_{h=1}-{\bar{Y}}_{h=2}}{{\left[\frac{Var\left(Y_{h=1}\right)}{n_{h=1}}+\frac{Var\left(Y_{h=2}\right)}{n_{h=2}}\right]}^{1/2}}$$

## 3 Results

### 3.1 Spatial-temporal distribution of NDVI

**Figure 2:**
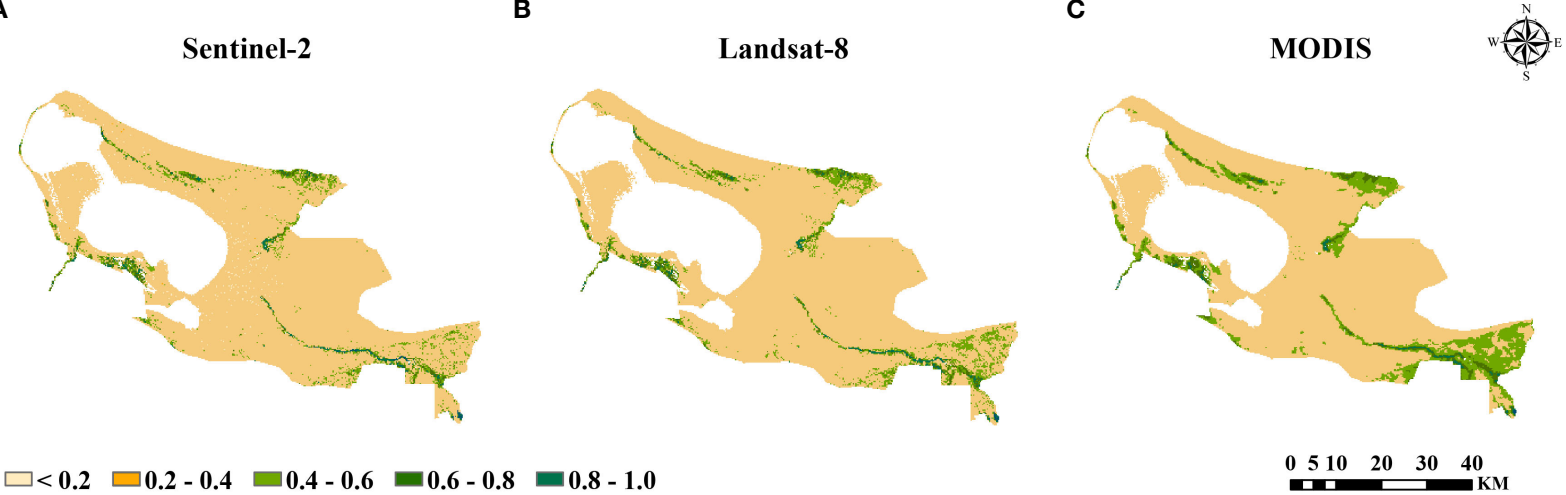
Normalized Difference Vegetation Index (NDVI) values at 250 m spatial resolution in **(A)** Sentinel-2, **(B)** Landsat-8, and **(C)** MODIS.

The original spatial resolutions of Sentinel-2, Landsat-8, and MODIS remote sensing images were 10 m, 30 m, and 250 m, respectively. Sentinel-2 and Landsat-8 remote sensing images were resampled to be consistent with the spatial resolution of the MODIS remote sensing images to obtain the average NDVI values of multi-source remote sensing from 2016 to 2022. ELWNNR vegetation growth distribution had significant spatial differences, all showed similar spatial variation patterns, with more areas of low values overall that presented low in the middle and high in the surrounding distribution characteristics (**Figure 2**). High vegetation coverage areas were mainly located in a small part of the Achiksu River and accounted for the smallest area of the whole region, with 0.14%, 0.05%, and 0.03%, respectively. Medium high vegetation coverage and medium vegetation coverage areas were mainly located around the Bortala River, the Kuitun River, the Achiksu River, and the Jinghe River, which accounted for 2.77%, 3.08%, and 3.61% of the area of the whole region, respectively. Medium low vegetation coverage areas were mainly located in the northern deserted area, the edge area of the Kuitun River, Hudong wetland, the Achiksu River, Toto River wetland, and part of the Bortala River confluence area, which accounted for 5.04%, 7.01%, and 12.97% of the whole area, respectively. The proportion of low vegetation coverage areas was the largest, and there were a large number of distributions in various parts of the whole area, and all of them were more than 80%, which were 92.05%, 89.86%, and 83.40%, respectively. The difference between the three remote sensing images was that, compared with the MODIS remote sensing images, the feature areas with a high vegetation index appeared clearer and occupied a larger area in Sentinel-2 and Landsat-8, while the lower vegetation areas occupied a smaller area and had a lower degree of refinement in the MODIS remote sensing images compared with the other remote sensing images. This indicated that the higher spatial resolution of remote sensing data was more effective in recognizing the fine features of remote sensing images, and it also played an auxiliary role in the recognition of low spatial resolution pixels.

### 3.2 Standard deviation and trend analysis of NDVI

**Figure 3:**
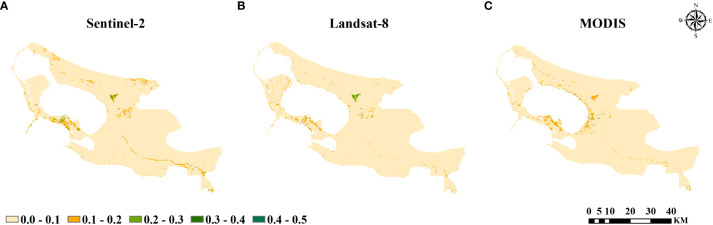
The standard deviation of NDVI in **(A)** Sentinel-2, **(B)** Landsat-8, and **(C)** MODIS.

The standard deviation of NDVI can reflect the degree of concentration as well as the stability of the data at a certain time and spatial scale. The standard deviation of NDVI was calculated based on Sentinel-2, Landsat-8, and MODIS remote sensing data. The spatial distribution of the standard deviation was similar and mostly lied between 0.0 and 0.1, and the fluctuation of the NDVI was relatively stable (**Figure 3**). The mean value of the standard deviation of Landsat-8 remote sensing images was the smallest at 0.023, and most of the standard deviation was close to 0.01, while that of Sentinel-2 and MODIS was slightly larger at 0.029, which indicated that the concentration of the Landsat-NDVI data was high and the representation was strong. This indicated that the Landsat-NDVI data were highly concentrated and representative.

**Figure 4:**
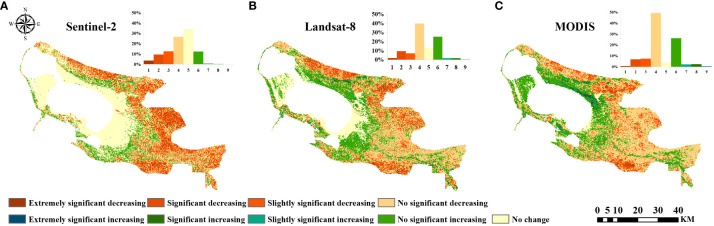
Significance Analysis in **(A)** Sentinel-2, **(B)** Landsat-8, and **(C)** MODIS.

Sen-MK trend analysis can reflect the changes of NDVI in a certain time space. The NDVI obtained by Sentinel-2, Landsat-8, and MODIS remote sensing imagery all showed similar spatial trends, and the area occupied by decreasing regions was larger than that occupied by increasing regions, which indicated that in the last seven years, ELWNNR vegetation showed a degradation trend (**Figure 4**). Overall, the proportion of NDVI obtained by Sentinel-2, Landsat-8, and MODIS sensors with a decreasing trend was 52.57%, 58.10%, and 64.66%, respectively, and the majority of the area was occupied by no significant decreasing. NDVI showed an increasing trend for 13.31%, 28.80%, and 31.32% of the total area, respectively, with no significant increasing occupied most of the area.

Specifically, the Sentinel-2, Landsat-8, and MODIS sensors differed in their sensitivity to NDVI trends. The no change area occupied a larger area in the Sentinel-2 remote sensing image, 20% more than the other datasets, which was due to the higher raw resolution of the Sentinel-2 remote sensing image and the sensor’s greater sensitivity to the area around the lake, which better identified the no change area around the lake. In Landsat-8 and MODIS remote sensing images, the trend change of no significant decreasing occupied the largest area, which was 10% more than other datasets, followed by no significant increasing occupied a larger area, which was 10% more than other datasets. This indicated that when the data with different spatial resolutions were resampled to the same spatial resolution, the lower spatial resolution decreased the larger area, while the higher original spatial resolution instead increased the smallest area.

In conclusion, among the three sensors mentioned above, the Sentinel-2 remote sensing image showed the smallest trend of vegetation greening, MODIS showed the largest trend of greening, and the Landsat-8 remote sensing image showed the middle degree of performance. This further reflected that the spatial distribution of NDVI trends in ELWNNR in the past 7 years was highly variable, and ecological problems still needed to be emphasized.

### 3.3 Comparative analysis of linear fitting of NDVI

**Figure 5:**
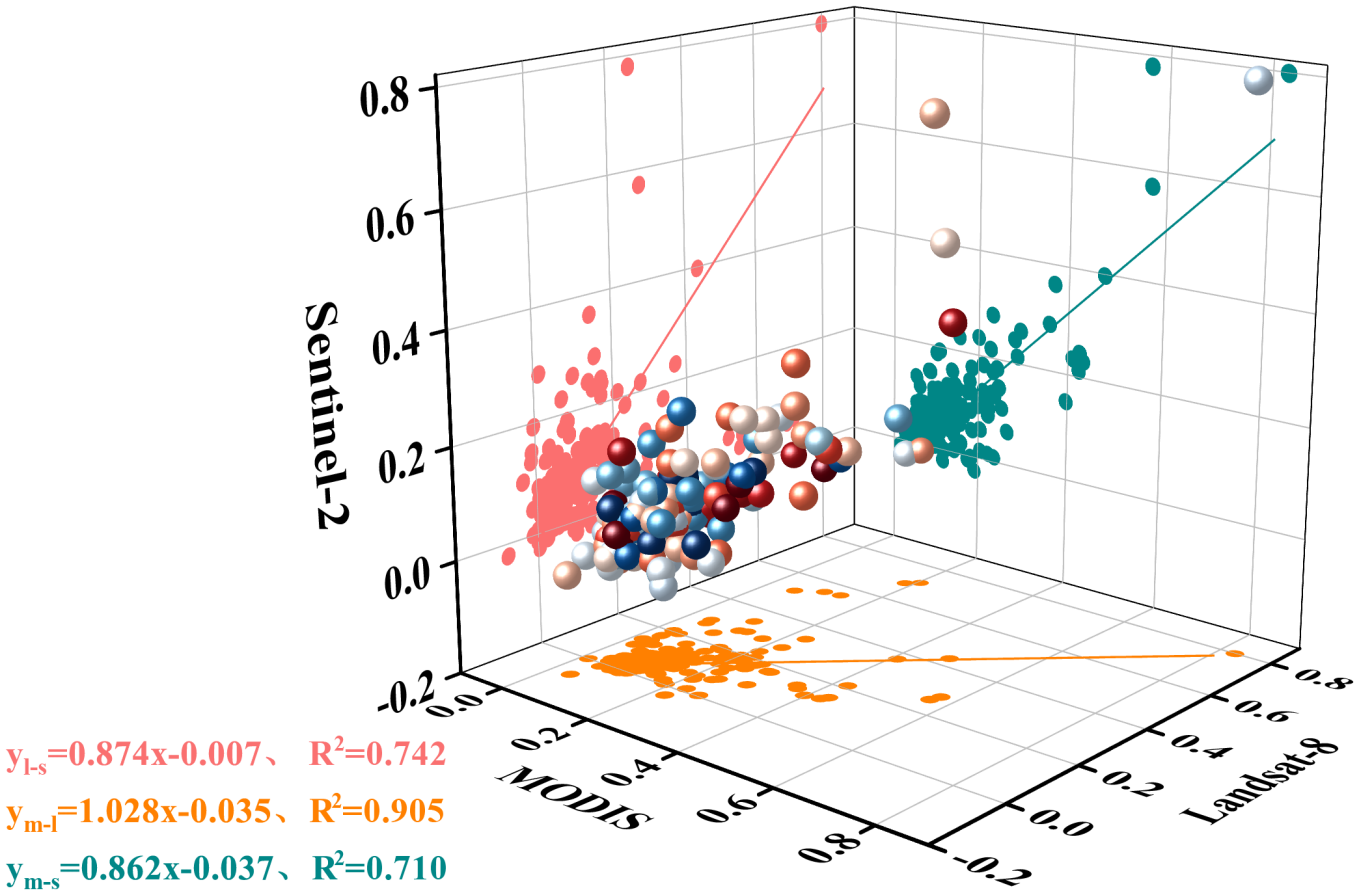
Comparative analysis of linear fitting of NDVI based on grid points for Sentinel-2, Landsat-8, and MODIS.

The fishing net was created by ArcGIS to form 266 fishing net points for linear fitting analysis to obtain the conversion equation and the coefficient of determination between the sensors. From the fitting of ELWNNR fishing net points, it can be seen that NDVI was most densely distributed between 0 and 0.4, with a better fitting effect, and sparsely distributed between 0.4 and 1, with a relatively poor fitting effect (**Figure 5**). The NDVI under the monitoring of Landsat-8 and MODIS sensors was closely aligned near the fitted curves (R^2 =^ 0.905, Pearson’s = 0.952) with high correlation, and the NDVI values under Landsat-8 monitoring were lower than those under MODIS monitoring because the coefficient of the conversion equation was greater than 1. The NDVI under Sentinel-2 and MODIS monitoring were relatively poorly fitted (R^2 =^ 0.710, Pearson’s = 0.842), the fit of NDVI values under Sentinel-2 and Landsat-8 sensor monitoring was intermediate (R^2 =^ 0.742, Pearson’s = 0.861), and NDVI values under Landsat-8 monitoring were higher than Sentinel-2 monitoring. Compared with Landsat-8 and MODIS, Sentinel-2 data had high resolution, which required multiple images to be spliced and processed, and the inconsistency in the acquisition time of remote sensing images will lead to a decrease in their correlation.

### 3.4 Analysis of the spatial pattern change of NDVI

**Figure 6:**
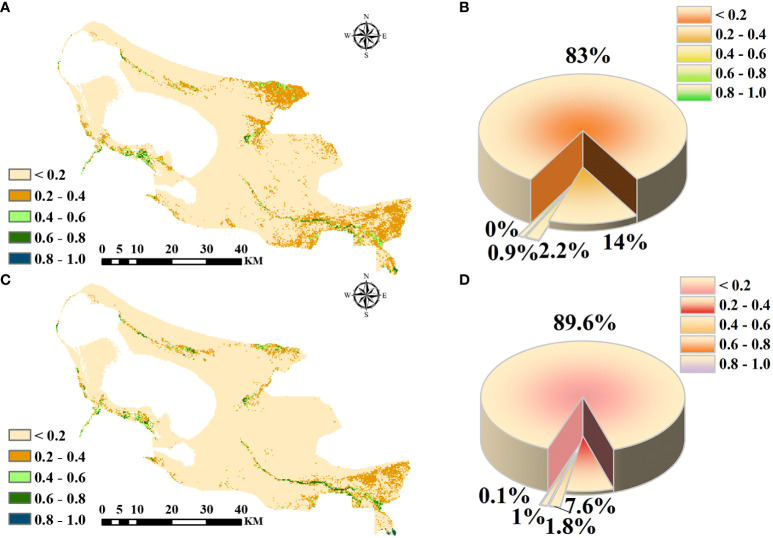
NDVI in 2016 **(A)**. Distribution of NDVI numbers as a percentage in 2016 **(B)**. NDVI in 2022 **(C)**. Distribution of NDVI numbers as a percentage in 2022 **(D)**.

The analysis of NDVI by Sentinel-2, Landsat-8, and MODIS remote sensing images showed that Landsat-8 data were more representative, so Landsat-8 remote sensing data were selected for vegetation dynamics analysis. 2016 and 2022 ELWNNR low vegetation cover areas and high vegetation cover areas accounted for 82.97% and 89.57%, 0.02% and 0.13%, respectively. Of the total area of the whole area, medium and low vegetation cover areas accounted for less than 15%, while medium and high vegetation cover areas accounted for less than 1%. The difference in vegetation cover in the whole region was obvious (**Figure 6**). Between 2016 and 2022, the area of medium low and medium vegetation cover areas showed decreasing trends; the area of medium high, high, and low vegetation cover areas showed increasing trends, but the amount of increase and decrease did not change much.

**Figure 7:**
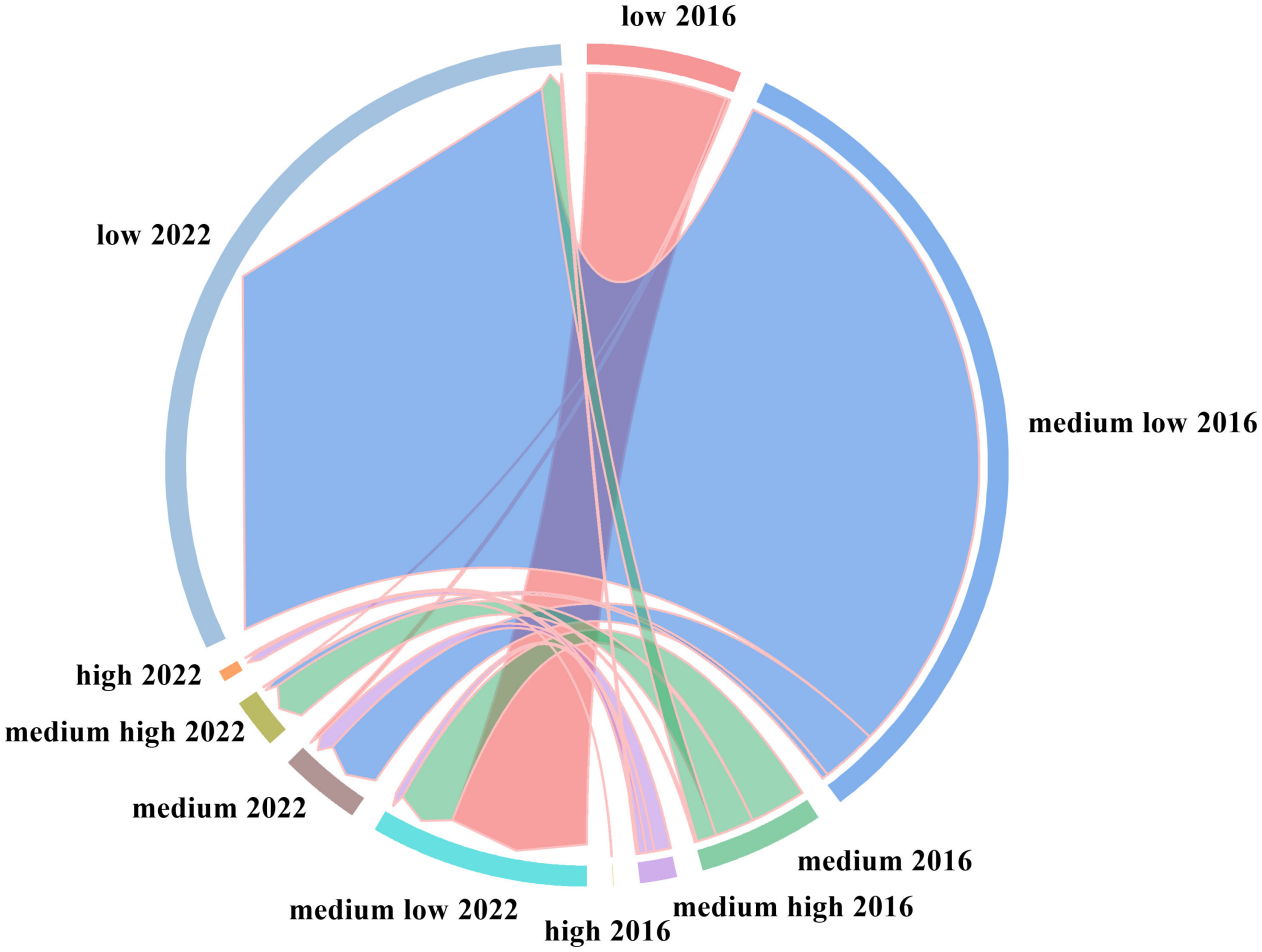
NDVI transfer changes from 2016 to 2022.

The spatial transfer matrix can quantitatively reflect the area change of different grades of NDVI as well as the transfer into and out of the area over a period of time, and it has been widely used in the study of vegetation cover. In this study, based on the 2016–2022 ELWNNR-NDVI for spatial distribution data statistics, the spatial change transfer matrix of NDVI of different grades was calculated (**Figure 7**). The transitions in NDVI grades were obvious. On the whole, NDVI in the whole region was characterized by a scarcity of high values and a wide range of low values.

Specifically, both the transferred-out and transferred-in areas were dominated by low vegetation cover and medium low vegetation cover, while 30.13 km^2^ of medium vegetation cover area was also transferred out. Prior to this, the vegetation ecosystem of ELWNNR was severely damaged, the restoration project was complicated, and the current vegetation situation was still not optimistic.

**Figure 8:**
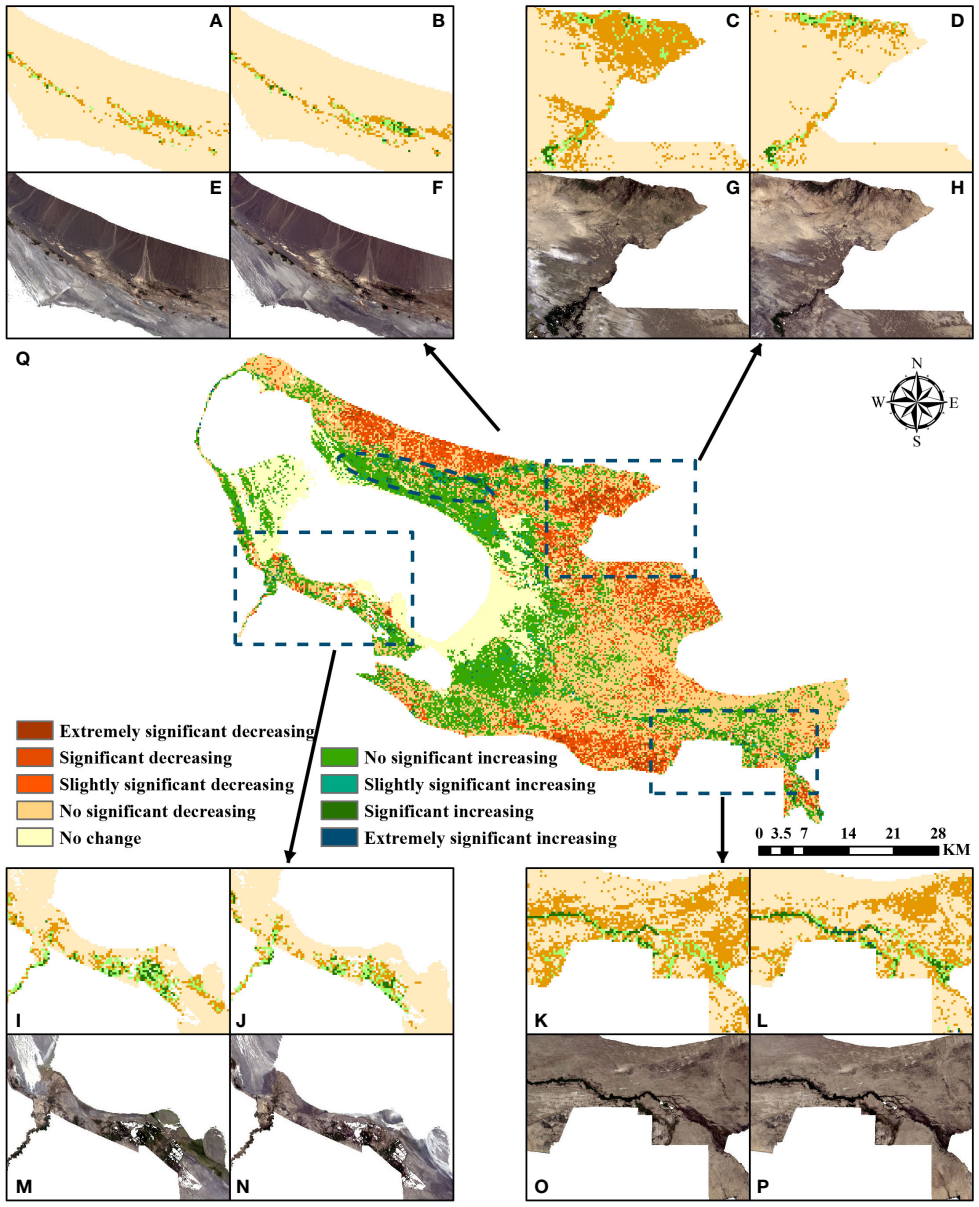
NDVI in 2016 **(A, C, I, K)**. NDVI in 2022 **(B, D, J, L)**. remote sensing image in 2016 **(E, G, M, O)**. Remote sensing image in 2022 **(F, H, N, P)**. NDVI significance analysis **(Q)**.

ELWNNR vegetation showed different degrees of improvement and degradation during the 7 years of change (**Figure 8**). There were areas of significant improvement and degradation in the northern part of the ELWNNR (**Figures 8A, B, E, F**), which were the KokeBastao area and the northern pre-mountain floodplain. In the area where houses were demolished after the ecological migration in KokeBastao, the population recovered naturally on a large scale, and the ecological environment was significantly improved by the crackdown on poaching and mining. 51.75 km^2^ of vegetation has been effectively improved, representing 65% of the area of the region. The changes in the NDVI vegetation in the last 7 years once again confirmed that the ecological migration was effective. In the northern pre-mountain floodplain, wind erosion had intensified, soil desertification was serious and unsuitable for vegetation growth, and the fragile ecological environment had led to vegetation degradation. 27 km^2^ of vegetation has been degraded, representing 33.8% of the area of the region. In addition, in the northeastern part of the area around the Kuitun River (**Figures 8C, D, G, H**), due to climate change in Xinjiang, water sources were decreasing, and the water source of the Kuitun River Basin was also decreasing or even cut off, which led to the destruction of the environment for the growth of the surrounding vegetation. Soil salinization and insufficient water sources cannot improve the ecological environment of the region, and the vegetation was obviously degraded. 100.91 km^2^ of vegetation has been degraded, representing 99.5% of the area of the region. In the southwestern part of the area near the northern part of the Jinghe River wetland and the area along the Bortala River (**Figures 8I, J, M, N**), the vegetation has increased and decreased. Previously, the vegetation was degraded due to fish and crab farming and human activities, but after the artificial closure of the area, the vegetation grew better due to the absence of human activities. However, due to the decrease in precipitation, the river cut-off, and the shrinkage of the lake area of Ebinur Lake, the water resource recharge has been reduced, the shrinkage of the lake area has changed the local climatic environment, and the surrounding area has been continuously sanded and salinized, resulting in the degradation of part of the vegetation. The area of increased and degraded vegetation in the district was 50.9 km^2^ and 41.6 km^2^, respectively. In the lower reaches of the Achiksu River (**Figures 8K, L, O, P**), the water around the river was rich in nutrients, and the soil texture was good, which was conducive to the growth of vegetation, and 54.6 km^2^ of vegetation along the river has been significantly improved. However, the vegetation in non-riverine areas was still seriously degraded, with 100.4 km^2^ of degraded vegetation accounting for 66.9% of the area of the district.

The above findings showed that both the degradation and improvement of the ecological environment were the result of the joint action of the natural environment and human activities. Through the comprehensive management of ecological restoration, although the overall degradation was evident, the local implementation of the protection of some areas of ecological recovery was obvious. Therefore, in the process of comprehensive management, the protection program should be proposed in real time in conjunction with the local natural environment, and a timely return visit should be made to update the restoration program.

### 3.5 Analysis of meteorological factors

**Figure 9:**
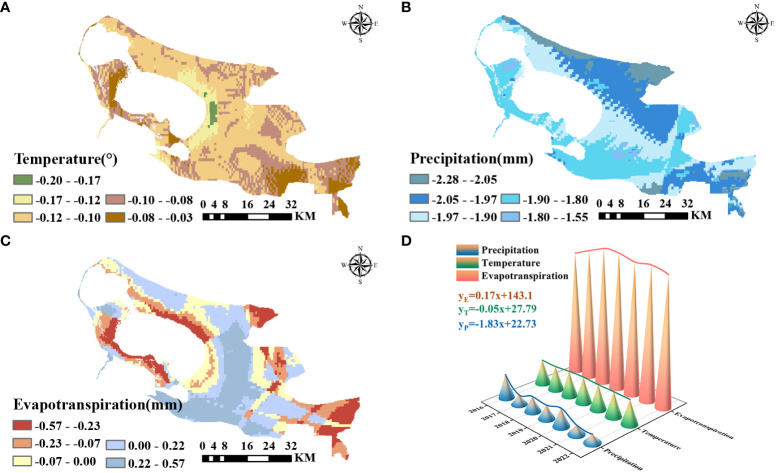
Temperature significance analysis from 2016 to 2022 **(A)**, precipitation significance analysis from 2016 to 2022 **(B)**, evapotranspiration significance analysis from 2016 to 2022 **(C)**, and time series analysis of temperature, precipitation, and evapotranspiration **(D)**.

Spatial trends of temperature, precipitation, and evapotranspiration were analyzed for the last 7 years in ELWNNR (**Figure 9**). Temperature showed an increasing and then decreasing trend from 2016 to 2022, with an overall decreasing trend (**Figure 9D**). The decreasing trend of temperature in the Toto River wetland and the upper edge of the Jinghe River wetland was smaller, with a rate of change of -0.08–0.03° per year. An area of significant temperature decline existed at the eastern edge of the ELWNNR lake center, with a rate of change of -0.20°–0.17° per year (**Figure 9A**). Overall, ELWNNR region-wide temperature showed a decreasing trend over the 2016–2022 period. In contrast to the temperature trend, ELWNNR precipitation has shown a decreasing trend in the last 7 years, with an increase but a more pronounced decrease in precipitation. In terms of spatial distribution, the northernmost and northeastern part of the region showed the highest decreasing trend with a decreasing rate of -2.28–2.05 mm per year (**Figure 9B**), while the central to northeastern part of the region showed a more pronounced decreasing trend with a decreasing rate of -2.05–1.97 mm per year. The evapotranspiration showed an increase and then a decrease in the time series, with an overall slight increase. The spatial distribution showed different areas of increase and decrease. A large area near the southeast corner of the Ebinur Lake shore showed a clear upward trend, with a rate of increase of 0.22–0.57 mm per year (**Figure 9C**), and the fastest rate of decrease was in the northern and southern parts of the Ebinur Lake shore and in the northeast and southeast regions of the whole region, with a rate of decrease of -0.57–0.23 mm per year. The rest of the rate of change was relatively smooth. Climate change had an effect on NDVI to some extent. According to statistical results from image elements, the greatest amount of vegetation with a degradation trend was found in the region, with a decreasing trend in precipitation of -2.05–1.97 mm per year, a decreasing trend in temperature of -0.12–0.10° per year, and an increasing trend in evapotranspiration of 0–0.22 mm per year. The largest amount of vegetation with an increasing trend was found in areas with a decreasing trend in precipitation of -1.90–1.80 mm per year, a decreasing trend in temperature of -0.12–0.10° per year, and an increasing trend in evapotranspiration of 0.22–0.57 mm per year.

### 3.6 Analysis of factors influencing NDVI

#### 3.6.1 Factor detection

**Figure 10:**
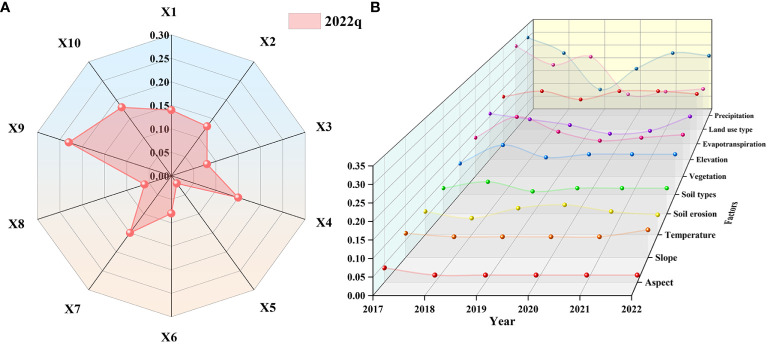
The q-values for factor detection in the ELWNNR in 2022 **(A)** and the changes in q-values for the ELWNNR factor from 2017 to 2022 **(B)**.

Factor detection can be used to reveal the degree of influence of each influencing factor on the NDVI. In this study, the influence of each factor on NDVI was determined by calculating the q-value of each factor (**Figure 10A**), and the descending order of the degree of influence was: precipitation (0.23) > evapotranspiration (0.18) > land use type (0.15) > elevation (0.15) > vegetation type (0.14) > soil type (0.13) > soil erosion (0.08) > slope (0.08) > temperature (0.06) > aspect (0.02). Among them, precipitation had the largest q-value with an explanatory power of 23%, which was much more influential than the other factors. Therefore, precipitation was the main driver of vegetation change in ELWNNR, water was one of the most important factors for vegetation development, and the study period was the growing season of vegetation, which promoted the growth of vegetation (Li et al., 2021). Evapotranspiration, land use type, elevation, vegetation type, and soil type had moderate explanatory power for the spatial distribution of NDVI, with explanatory power greater than 10%. While soil erosion, slope, temperature, and aspect were smaller and the explanatory power was below 10%, the explanatory power was less than 10% and could have an effect by interacting with other imaging factors.

From the temporal trends of the influencing factor q-values (**Figure 10B**), aspect, temperature, evapotranspiration, and soil type showed an increasing trend from 2017 to 2022. Aspect, soil erosion, vegetation type, elevation, land use type, and precipitation showed a decreasing trend. Among them, aspect, slope, temperature, soil erosion, evapotranspiration, and soil type q-values changed more smoothly, with rates of change of -0.29%, 0.14%, 0.06%, -0.14%, 0.23%, and 0.2%, respectively. Vegetation type and elevation q-values changed more significantly, with rates of -0.54% and -0.57%, respectively. The land use type q-value of NDVI decreased faster, at -3.54%. Natural factors dominated ecological and environmental restoration, and anthropogenic closure and protection were only auxiliary means. And among them, the fluctuation of precipitation was large, but its rate of change was smaller than that of land use types, with the q-value showing a decreasing trend in 2017–2019 and the opposite in 2019–2022, with an overall rate of change of -0.77%, which was inextricably linked to the trend of precipitation change in the six years period of first increasing and then decreasing. This suggested that ELWNNR-NDVI changes were related to environmental factors, and changes in environmental conditions had a profound impact on the ecological environment, leading to spatial differentiation in ELWNNR-NDVI.

#### 3.6.2 Ecological detection

Ecological detection can reflect whether there were significant differences in the effects of each detected factor on the spatial distribution of NDVI. There was no significant difference between vegetation type and soil type, elevation, land use type, elevation and soil type, land use type, land use type and evapotranspiration (detection value of N), and there was a significant difference in the effect of all other factors on NDVI (detection value of Y). Specific values and significance will be expressed in **Table 2**. The results of the factor detection showed that precipitation was the dominant factor leading to changes in NDVI, and the ecological detection results further proved that the effect of precipitation was greater than that of other factors.

**Table 2:** Ecological detection of factors.

Factors	X1	X2	X3	X4	X5	X6	X7	X8	X9	X10
X1										
X2	N									
X3	Y	Y								
X4	N	N	Y							
X5	Y	Y	Y	Y						
X6	Y	Y	N	Y	Y					
X7	N	Y	Y	N	Y	Y				
X8	Y	Y	Y	Y	Y	Y	Y			
X9	Y	Y	Y	Y	Y	Y	Y	Y		
X10	Y	Y	Y	Y	Y	Y	N	Y	Y	

Statistical significance of detection factors (95% confidence level).

#### 3.6.3 Interaction detection

**Figure 11:**
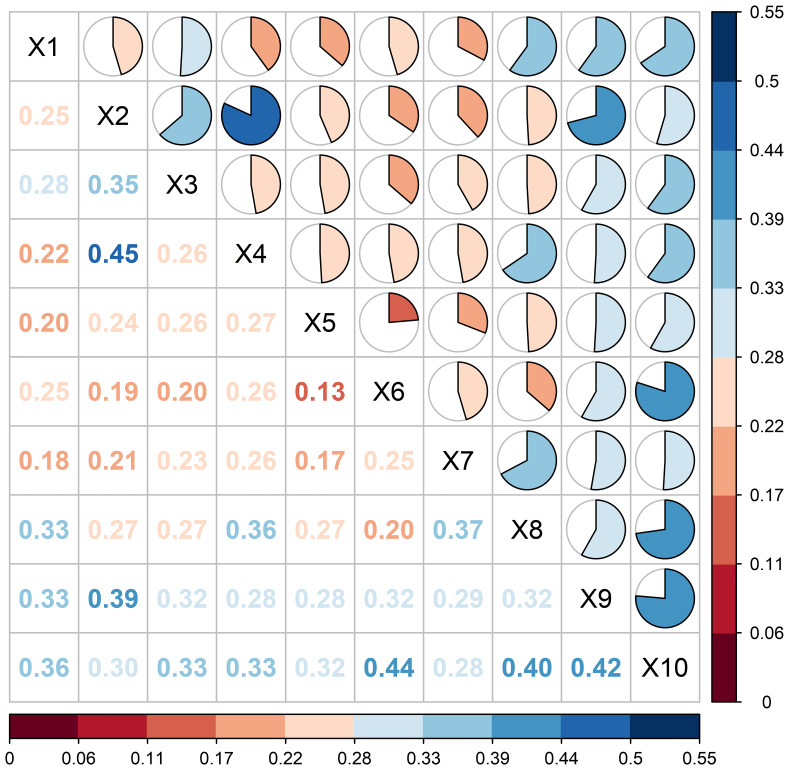
ELWNNR factor interaction detection.

Interaction detection mainly referred to detecting the interaction of different factors on the changes of vegetation NDVI, analyzing whether the explanatory power of the dependent variable NDVI will be increased or weakened, or whether the effects on vegetation NDVI were independent of each other, and further evaluating the differences between single and two-factor. The interactions showed that each factor interacted with each other on the effect of NDVI (**Figure 11**). 29% of the interactions showed a two-factor enhancement relationship, and 71% of the driver interactions showed a nonlinear enhancement relationship. The largest interaction was between soil type and elevation, with a q-value of 0.45, which had the strongest explanatory power for spatial differentiation among all factors. The interactions of evapotranspiration and soil erosion, temperature, and precipitation similarly exceeded 40%, with 44%, 40%, and 42%, respectively. Land use type and soil erosion showed a nonlinear enhancement, and the rest were two-way enhancements. The q value of the interaction between the driving factors was higher than that of any single factor, and the statistical analysis showed that the interaction between the factors showed two-factor enhancement and nonlinear enhancement, and there was no factor acting independently of each other, so that the driving factors would increase the influence on the stability of the NDVI when they acted together. In summary, the difference between the ecological and interactive results showed that the spatial variability of ELWNNR-NDVI was not a simple superposition of factors but a result of the mutual enhancement of multiple factors.

#### 3.6.4 Risk detection

Risk detection can explore the type or range of factors that were most adapted for vegetation growth and test for statistical significance at the 95% confidence level. The appropriate range or type of factor was crucial for vegetation growth, and different ranges or types of factors had a significant effect on NDVI, with higher NDVI indicating that the eigenvalues of the factors were more suitable for vegetation growth. NDVI varies greatly among the factors (**Table 3**).

**Table 3:** The suitable limits of the natural factors (95% confidence level).

Factors	Suitable types or range	NDVI
Vegetation	Broad-leaved forest	0.180
Soil types	Water becomes soil	0.206
Slope (°)	0.74–1.14	0.173
Elevation (m)	423–480	0.844
Aspect(°)	0–22.5、337.5–360	0.127
Soil erosion	Mild hydraulic erosion	0.280
Land use type	Crops	0.844
Temperature(°C)	25.4–25.8	0.178
Precipitation(mm)	11.8–13.6	0.206
Evapotranspiration(mm)	131–138	0.238

NDVI had different values for different land use types (water bodies, trees, submerged plants, crops, built-up areas, bare land, and pasture), and was greatest in crop areas at 0.844. NDVI increased with increasing precipitation and was greatest at 0.206 when precipitation was between 11.8 mm and 13.6 mm. NDVI was at its maximum at 0.178 when the temperature was between 25.4°C and 25.8°C. The evapotranspiration reached 0.238 when the evapotranspiration was between 131 mm and 138 mm.

NDVI also varies with vegetation type, soil type, and soil erosion. When the vegetation type was broad-leaved forest, the NDVI was more than 0.180. When the soil type was water becomes soil, the soil had a strong fertilizer-holding capacity, which was conducive to the growth of vegetation, the NDVI was 0.206. Soil erosion also affects the NDVI to some extent, with an NDVI of 0.280 for mild hydraulic erosion. With the change in elevation, the NDVI also underwent a certain change. When the elevation was 423–480 m, the altitude was relatively suitable and favorable for the growth of vegetation, and the NDVI was 0.177. NDVI was 0.173 and 0.127 for slopes between 0.74° and 1.14° and for aspects of 0° to 22.5° and 337.5° to 360°, respectively.

## 4 Discussion

### 4.1 Analysis of trends in vegetation change

ELWNNR, within the largest saltwater lake in Xinjiang, has some unique features. The wetland is surrounded by abundant natural medicinal herbs, vegetation, and halophytes, which are important for the entire economic zone of the northern slope of Tianshan Mountain (Li et al., 2019b). This study used Sentinel-2, Landsat-8, and MODIS remote sensing data to quantitatively analyze the trend of vegetation in ELWNNR, all of which showed good consistency and a similar spatial distribution, that is, low in the middle and high in the surroundings. The NDVI calculated from MODIS remote sensing data was higher than the values calculated from Landsat-8 and Sentinel-2 remote sensing data. The MODIS image adopted the maximum value synthesis method, and the mixing effect was more obvious with the low original resolution, which led to the high NDVI values. Meanwhile, the Sentinel-2 remote sensing image had a higher original resolution, and the sensor was more sensitive to the area around the lake, which can recognize the unchanged area around the lake, thus leading to the low NDVI values. The standard deviation was mostly between 0.0 and 0.1, and the fluctuation in NDVI was relatively stable. The linear fitting coefficients of Landsat-8 and MODIS, Sentinel-2 and MODIS, and Sentinel-2 and Landsat-8 were 0.952, 0.842, and 0.861, respectively. As mentioned before, the differences in spatial resolution and waveband of multi-source remote sensing data lead to differences in the NDVI generated from each data and the fitting effect between them. Combining the similar results of previous studies and ours, it can be seen that using Landsat-8 remote sensing data to analyze the vegetation changes in ELWNNR has better results. Some previous studies obtained similar results to ours. Sentinel and Landsat fitted better, and the fit with MODIS was relatively low (Hu et al., 2022; Wang et al., 2022). Landsat slightly outperformed the other sensors in all performance metrics (Sajadi et al., 2021). The correlation between MODIS and SPOT was better on a national scale, while the correlation between dryland GIMMS and MODIS was better (Zhu et al., 2019). A comparison of differences and functional relationships between two different sets of sensors, the Advanced Very-High-Resolution Radiometer (AVHRR)-NDVI and MODIS-NDVI, has also been done (Zhang et al., 2016). Research and analysis of the Hanjiang River vegetation changes by using three sensors, AVHRR, MODIS, and SPOT, showed that MODIS-NDVI was more similar to SPOT-NDVI, and MODIS-NDVI was able to clearly distinguish crops and reflect the diversity of ground vegetation (Yuan and Maichun, 2015). Different remote sensing data have different suitability for different study areas, and the most suitable data should be selected.

### 4.2 Analysis of NDVI under embargoed protection

The ecological recovery of the areas with localized implementation of closure and protection was obvious, which suggested that the prohibition of human activities was an effective measure. The trend of vegetation change showed obvious spatial heterogeneity. In the region of KokeBastao, where ecological migration and human activities have been greatly reduced, the vegetation has recovered on a large scale, and the ecological environment has been significantly improved. The northern part of the Jinghe wetland and the area along the Boltara River showed a significant increase in vegetation after the ban on artificial farming without human activities. The Akobastao and Achiksu rivers had sufficient hydrothermal conditions, and the hydromorphic soils were highly fertile and suitable for vegetation growth (Gao et al., 2022). By contrast, the prehill floodplain area has experienced increased soil desertification and obvious vegetation degradation due to increased wind erosion. The area around the Kuitun River was significantly degraded because of the river cutoff, the reduction in the lake area, and the salinization around the lake (Wang et al., 2021a). Vegetation was closely related to water conditions, and the magnitude of water resources affects vegetation growth. The increase in temperature and precipitation was positively correlated with the increase in aboveground biomass (Yan et al., 2015). The average precipitation and temperature in ELWNNR showed a decreasing trend year by year, and the decreasing trend of precipitation was significantly larger than the decreasing trend of temperature; meanwhile, evaporation was fast, which will lead to localized drying and inhibit the growth of vegetation (Liu et al., 2014). The above trend of vegetation change indicated that the vegetation of ELWNNR tended to degrade in the context of the natural environment. Nevertheless, after the artificial relocation, the vegetation in the area protected by the artificial closure improves obviously, and the ecological environment was effectively enhanced. Thus, the implementation of total closure can improve the ecological environment of the protected area, and the current measures were effective. By contrast, the Zhangye Heihe Wetland Reserve, which was also an arid area, suffers from area shrinkage and vegetation degradation and requires improving vegetation cover under suitable conditions (Li et al., 2019a). Vegetation testing of two wetland reserves in the Dunhuang Yangguan Nature Reserve in Gansu found that soil moisture availability had the greatest impact on vegetation in extremely arid areas, with precipitation also having a partial effect (Pan et al., 2018). The Tarim River Basin in the arid zone has shown an increasing trend in vegetation in recent years, and part of the vegetation degradation was related to downstream river breaks, overgrazing, and intensified human activities, which was similar to the causes of vegetation degradation in this study area (Wang et al., 2023). According to a study of vegetation and rainfall in the semi-arid Nylsvley Wetland Reserve in South Africa, many years of drought and human activities have resulted in a negative pattern of NDVI (Murungweni et al., 2020). On the contrary, the vegetation of the Lean wetland in the humid zone was in good condition and continues to be stable. Part of the vegetation degradation was anthropogenic, mainly for economic development and construction (Ying et al., 2019). Most of the vegetation in the Shengjin Lake Wetland Reserve in the humid zone tends to improve with local degradation. The nature factor showed a significant positive effect, while the social factor had an opposite effect (Yang et al., 2016). Vegetation in the Fujian Zhangjiangkou Mangrove Wetland Reserve in the humid zone was generally improving, but the surrounding residents rely heavily on mudflats; the relationship between humans and vegetation needed to be harmonized to promote mangrove wetland restoration (Zhao et al., 2011).

The results of previous studies and the present study showed that ecological changes in arid, semiarid, and humid zones were the result of the combined effects of the natural environment and human activities. Localized improvement of the ecological environment can be achieved by human-induced closure, which needed to be adapted to local conditions and strictly managed. The overall vegetation recovery depended mainly on the long-term shaping of the natural environmental conditions, with the use of closure as a supplementary measure. Therefore, the implementation of closed protection can be an effective way to improve the ecological environment of protected areas. Under the national management policy, to strengthen the idea of ecological civilization construction in protected areas, local governments should continue to carry out ecological restoration and scientific management guided by green development.

### 4.3 Analysis of NDVI driving factors

**Figure 12:**
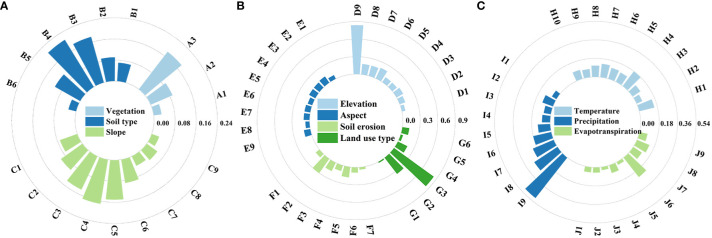
Risk detector results for each factor. **(A)** Vegetation, soil type, and slope; **(B)** elevation, aspect, soil erosion, and land use type; **(C)** temperature, precipitation, and evapotranspiration.

Trends in ELWNNR vegetation and the causes of change have been confirmed, but the drivers needed to be explored further. We used geographical detectors to investigate the effects of factors and interactions between factors on NDVI and to determine the most appropriate characteristics of the factors. Precipitation was the main driver of vegetation change in ELWNNR and had the strongest explanatory power for NDVI, exceeding 20%. It was followed by evapotranspiration, land use type, elevation, vegetation type, and soil type, which had moderate explanatory power for the spatial distribution of NDVI, with explanatory power greater than 10%. Meanwhile, soil erosion, slope, air temperature, and slope orientation all had less explanatory power, with explanatory power below 10%. Accordingly, after the implementation of ecological management projects and the change of the natural environment in recent years, the effect of vegetation restoration in the area of anthropogenic protection is very significant. The results of the interaction analysis showed that the ecological restoration project was a complex, multifactorial, and integrated process. No single factor can have absolute explanatory power for NDVI, the value of the interaction between the driving factors was higher than that of any single factor, and the interaction between the factors showed a two-factor and nonlinear enhancement relationship. No factor acted independently, so the driving factors will increase their influence on the stability of NDVI when they act together. The different factors were explicitly discussed in the risk detection to further understand their specific distribution values (**Figure 12**). The greater the values of elevation and precipitation factors, the higher the NDVI values, indicating that elevation and precipitation were positively correlated with NDVI. The region with lower elevation had high soil salinity, a low water table, and an uneven distribution of temperature and precipitation due to topographic factors, which results in a clear separation of high and low vegetation indices. Among the land use types, crops corresponded to high vegetation cover, flooded vegetation was moderate, and the rest was low. Among the vegetation types, broadleaf forests had the highest NDVI values, followed by mixed coniferous broadleaf forests and coniferous forests. In ELWNNR, years of ecological migration and afforestation increased the amount of vegetation. Precipitation and temperature conditions were relatively good under suitable elevation conditions (Chen et al., 2010). The most suitable altitude in this study was 423–480 m. As the elevation increased, the natural conditions changed, as did the NDVI. Slope orientation usually changes the hydrothermal conditions for vegetation growth by altering surface runoff and affecting sunlight intensity, and shady slopes were more suitable for vegetation growth than sunny slopes with abundant water and higher nutrient content (Liu and Wang, 2013). However, the effects of slope and orientation were relatively small in our study. In arid and semiarid zones, precipitation and temperature were the main factors in vegetation changes (Liu et al., 2016). In most of the northwestern region, vegetation was sensitive to hydrothermal conditions, and climate change can greatly affect vegetation (Jiao et al., 2021). In plains, anthropogenic impacts on vegetation were significant (Sun et al., 2015). In the Inner Mongolia Plateau in northern China, precipitation became the strongest factor inhibiting vegetation growth (Sun et al., 2021). In summary, vegetation dynamics are affected by a combination of factors, not a single anthropogenic factor. Factors and anthropogenic factors interact to make differences in vegetation change.

### 4.4 Shortcomings and prospects

In this study, Sentinel-2, Landsat-8, and MODIS remote sensing data were utilized to quantitatively monitor the vegetation restoration in ELWNNR, and the results of their monitoring showed consistency in overall spatial distribution and temporal trend. However, in the comparison of multi-source remote sensing data, UAV (Unmanned Aerial Vehicle) imagery, multispectral, and hyperspectral data have good performance for vegetation information expression, which can be further analyzed and compared. For vegetation indices, we chose NDVI, but given the wide variety of vegetation indices in different bands, future studies can add more vegetation indices for comparison. In particular, the red-edge vegetation index performs well in sparsely vegetated areas, and the accuracy of the data can be verified by combining it with field surveys, which is one of the directions of our future research. In the balanced relationship between ecological protection and economic development, the relationship between the ecological benefits brought by enclosing protected areas and the economic benefits brought by allowing human production deserves in-depth exploration. Comprehensive closure protection improves the ecological environment to a certain extent, and we can consider introducing activities that can generate economic benefits to enhance the local Gross Domestic Product (GDP) and promote the optimization of the ecological environment through the joint management of enterprises and the government.

## 5 Conclusions

We used the comparative analysis of Sentinel-2, Landsat-8, and MODIS multi-source remote sensing data, combined with the Mann-Kendall trend test and geographical detector model, to explore in depth the trend of NDVI and the influence of each influencing factor on NDVI from 2016 to 2022. We explored whether the total closure protection measures can provide help for ecological recovery in the protected area. Specific conclusions are presented below:

(1) The spatial distributions of NDVI based on Sentinel-2, Landsat-8, and MODIS data were similar, showing a low distribution in the middle and a high distribution around, with stable changes. Among the different data sources, MODIS-NDVI had the highest value, while Sentinel-NDVI had the lowest value. The coefficients of linear fit for Landsat-8 and MODIS, Sentinel-2 and MODIS, and Sentinel-2 and Landsat-8 were 0.952, 0.842, and 0.861, respectively.
(2) The 2016–2022 ELWNNR vegetation showed a general trend of degradation, with significant improvement in localized areas of anthropogenic protection. The whole region showed the characteristics of few and sparse high values and many and extensive low values. Changes in the ecological environment are the result of the joint role of man and nature. Human intervention will affect the local ecological change of the environment, but the overall ecological changes are still dominated by natural environmental factors.
(3) The degree of influence of each factor on NDVI was precipitation > evapotranspiration > land use type > elevation > vegetation type > soil type > soil erosion > slope > temperature > aspect. The interaction of each factor on NDVI showed two-way and nonlinear enhancement relationships, and each driver increased the influence on vegetation cover stability when they acted together.

Therefore, the implementation of total closure can be a proven way to promote ecological restoration. However, improvement methods must be established under suitable natural conditions and combined with policies to produce positive effects. Future research can analyze the balance between ecological protection and economic development together with future trends to provide reasonable and effective recommendations for ecological environment management and restoration in wetland reserves in arid areas.

## Data availability statement

The datasets presented in this study can be found in online repositories. The names of the repository/repositories and accession number(s) can be found below: https://earthexplorer.usgs.gov/, http://www.gscloud.cn/, https://scihub.copernicus.eu/dhus/#/home, http://www.gscloud.cn, https://www.resdc.cn/, https://data.tpdc.ac.cn/homearcgis.com.

## Author contributions

NX: Conceptualization, Data curation, Formal analysis, Investigation, Methodology, Project administration, Software, Validation, Writing – original draft, Writing – review & editing. YT: Data curation, Formal analysis, Investigation, Methodology, Project administration, Software, Validation, Writing – original draft. MT: Software, Writing – review & editing. WQ: Conceptualization, Writing – review & editing. ZX: Formal analysis, Writing – review & editing. BZ: Project administration, Writing – review & editing. YX: Software, Writing – review & editing. YM: Funding acquisition, Writing – review & editing.
