# Supplementary material for: Monitoring and evaluation of vegetation restoration in the Ebinur Lake Wetland National Nature Reserve under lockdown protection
Source: Front Plant Sci. 2024 Apr 18;15:1332788. doi: 10.3389/fpls.2024.1332788 (PMC11063322; doi:10.3389/fpls.2024.1332788)
Supplement: Supplementary file 1 [file DataSheet_1.pdf]

Supplementary Material

Monitoring and evaluation of vegetation restoration in the Ebinur Lake Wetland National Nature Reserve under lockdown protection

Yuqian Tang[1,2,3], Nan Xia[1,2,3,4,*,†], Mengying Tang[1,2,3], Weilin Quan[1,2,3], Zhanjiang Xu[1,2,3], Bowen Zhang[1,2,3], Yuxuan Xiao[1,2,3]

*** Correspondence:**

Corresponding author at: 777 Huarui Street, Shuimogou District, Urumqi, Xinjiang, China.

E-mail address: xn_gis@xju.edu.cn (N. Xia).

†These authors contributed equally to this work and share first authorship.

1 Supplementary Figures and Tables

1.1 Supplementary Figures

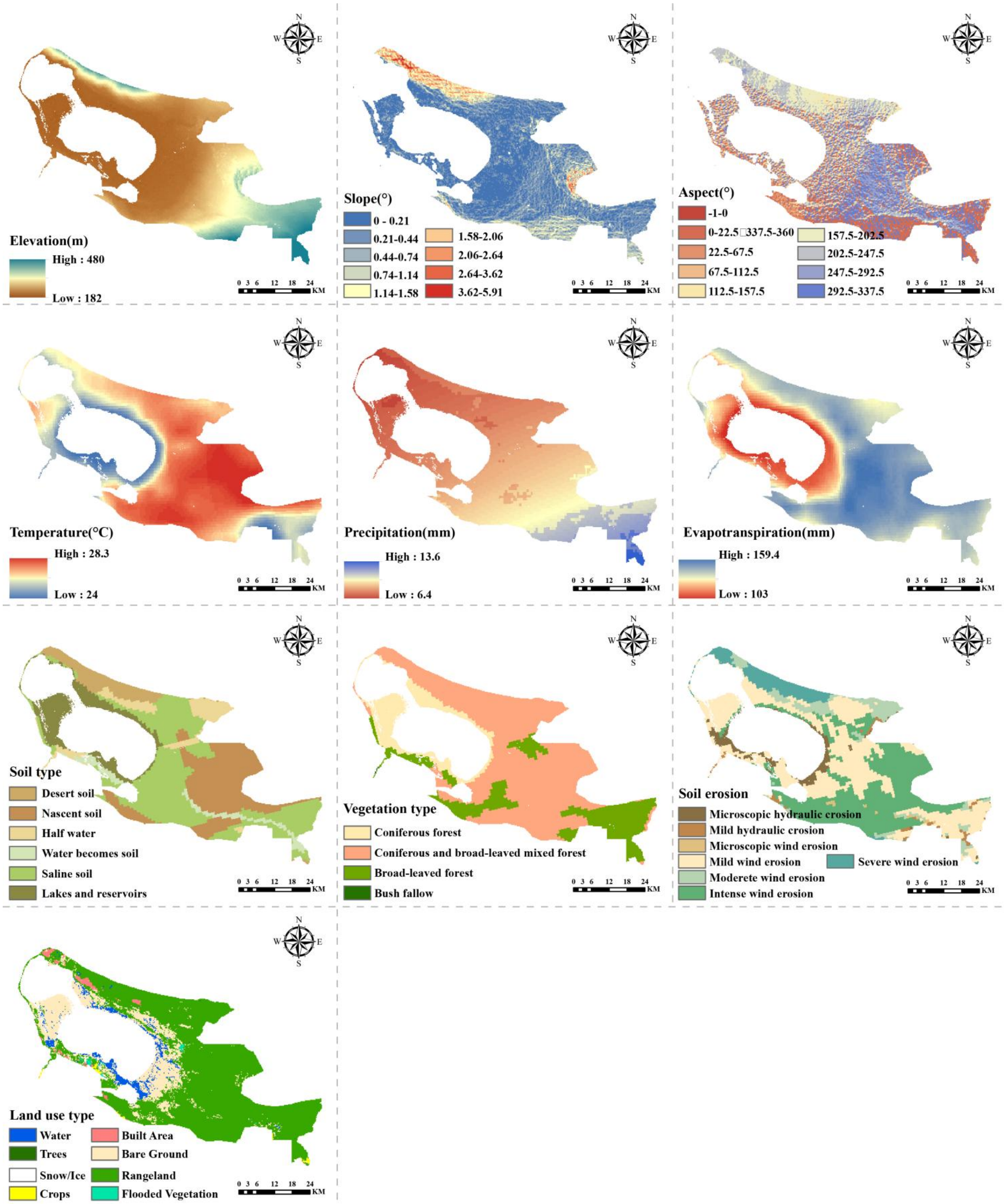

Figure S1 Impact Factors.

1.2 Supplementary Tables

Table S1 Detection Factors

Factors	Code	Classification
Vegetation	X1	7
Soil type	X2	6
Slope	X3	9
Elevation	X4	9
Aspect	X5	9
Soil erosion	X6	7
Land use type	X7	7
Temperature	X8	10
Precipitation	X9	9
Evapotranspiration	X10	9

Table S2 Criteria for interaction detection

Criteria for judgment	Interaction
$q(X1\cap X2)<Min(q(X1),q(X2))$	Nonlinear attenuation
$Min(q(X1),q(X2))<q(X1\cap X2)<Max(q(X1),q(X2))$	Single factor nonlinear attenuation
$q(X1\cap X2)>Max(q(X1),q(X2))$	Double factor enhancement
$q(X1\cap X2)=q(X1)+q(X2)$	Independence
$q(X1\cap X2)>q(X1)+q(X2)$	Nonlinear enhancement
